# Transient Ischemic Attack and Ischemic Stroke in Danon Disease with Formation of Left Ventricular Apical Thrombus despite Normal Systolic Function

**DOI:** 10.1155/2017/6576382

**Published:** 2017-09-20

**Authors:** Takeshi Tsuda, Amanda J. Shillingford, Jane Vetter, Vinay Kandula, Badal Jain, Joel Temple

**Affiliations:** ^1^Nemours Cardiac Center, Nemours/Alfred I. duPont Hospital for Children, Wilmington, DE 19803, USA; ^2^Department of Medical Imaging, Nemours/Alfred I. duPont Hospital for Children, Wilmington, DE 19803, USA; ^3^Division of Neurology, Nemours/Alfred I. duPont Hospital for Children, Wilmington, DE 19803, USA

## Abstract

Danon disease is a rare X-linked dominant skeletal and cardiac muscle disorder presenting with hypertrophic cardiomyopathy, Wolf-Parkinson-White syndrome, skeletal myopathy, and mild intellectual disability. Early morbidity and mortality due to heart failure or sudden death are known in Danon disease, more in males than in females. Here, we present a 17-year-old female adolescent with Danon disease and severe concentric hypertrophy with normal left ventricular (LV) systolic function, who has been complaining of intermittent headache and weakness for about 3 years, initially diagnosed with hemiplegic migraine. Subsequently, her neurological manifestation progressed to transient ischemic attack (TIA) and eventually to ischemic stroke confirmed by CT scan with 1-day history of expressive aphasia followed by persistent left side weakness and numbness. Detailed echocardiogram for the first time revealed a small LV apical thrombus with unchanged severe biventricular hypertrophy and normal systolic function. This unexpected LV apical thrombus may be associated with a wide spectrum of neurological deficits ranging from TIA to ischemic stroke in Danon disease. Possibility of cerebral ischemic events should be suspected in Danon disease when presenting with neurological deficits even with normal systolic function. Careful assessment for LV apical thrombus is warranted in such cases.

## 1. Introduction

Danon disease is a rare genetic disorder presenting with progressive cardiac hypertrophy, Wolf-Parkinson-White (WPW) syndrome, skeletal myopathy, and mild intellectual disability [1, 2]. Rapid deterioration and early sudden cardiac death have been reported in this disorder mostly due to congestive heart failure and/or ventricular arrhythmia in association with concentric ventricular hypertrophy that phenotypically resembles hypertrophic cardiomyopathy (HCM) [3–5]. Affected male patients tend to develop an earlier and more severe clinical course than female patients [2, 6], but female patients occasionally present with early progressive course [7–9]. Here, we present a 17-year-old female adolescent with Danon disease with severe HCM who developed infrequent neurological episodes diagnosed as recurrent hemiplegic migraine over 3 years, followed by possible transient ischemic attack (TIA) over the past 6 months and finally ischemic stroke. At the time the stroke was identified by positive CT scan, a thrombus in the left ventricular (LV) apex was also detected by an intensive echocardiographic investigation. This intermittent but progressive neurological deficit in Danon disease is discussed in conjunction with a role of microembolism in association with the formation of intracardiac thrombus in otherwise normally contracting hypertrophic LV.

## 2. Case Report

A 17-year-old female adolescent was admitted to our hospital because of 1-day history of expressive aphasia followed by persistent left-side weakness and numbness. At 8 years of age, she was noted to have a remarkable concentric, but nonobstructive, biventricular hypertrophy on echocardiogram when she was referred to our center for evaluation of chest pain and palpitation. Initial ECG showed short PR interval and severe LV hypertrophy (LVH) with strain pattern but without delta wave. ECG findings of WPW syndrome became evident at 10 years of age. Her family history was not entirely clear, but neither biological parent has Danon disease. At 12 years of age, she underwent cardiac catheterization and an electrophysiological study (EPS), which revealed elevated LV end diastolic pressure (LVEDP) 15 mmHg, elevated RV end diastolic pressure (RVEDP) 10 mmHg, normal cardiac index (CI) 4.0 L/min/m^2^, and normal pulmonary vascular resistance (Rp 1.9 Wood unit/m^2^). A right anterior accessory pathway was identified and was ablated with cryotherapy. Genetic testing confirmed the diagnosis of Danon disease with lysosome-associated membrane protein 2 (LAMP2) mutation (Va1310Ile mutation). During this admission, she was found to have frequent premature ventricular complexes (PVCs) and occasional multifocal nonsustained ventricular tachycardia (VT). She was started on atenolol, but she was not compliant with the medication. At 13 years and 4 months of age, she underwent implantable cardioverter defibrillator (ICD) implantation for frequent nonsustained polymorphic VT. Neurologically, she reported having monthly headaches for many years associated with nausea, usually during her menstrual cycles. Since nearly 15 years of age, she has had intermittent transient episodes of blurry vision and left arm and left face numbness lasting 10 minutes, followed by headaches lasting approximately 20 minutes. At 15 years and 9 months of age, she was admitted to our hospital for overnight observation of transient headache, photophobia, right-sided weakness and numbness, and abdominal pain. She was diagnosed with hemiplegic migraine with aura upon negative head CT scan done 12 hours after the event. Her medication was switched to nadolol. Over the course of 6 months prior to the current ischemic stroke, she was admitted twice for transient mild left side hemiparesis and difficulty in verbalizing words. On both occasions, she was discharged home on the following day since she was back to her baseline status with negative head CT scan and CT angiography. Because of the high possibility of transient ischemic attack (TIA), she was placed on daily aspirin, which she did not take regularly.

**Figure 1:**
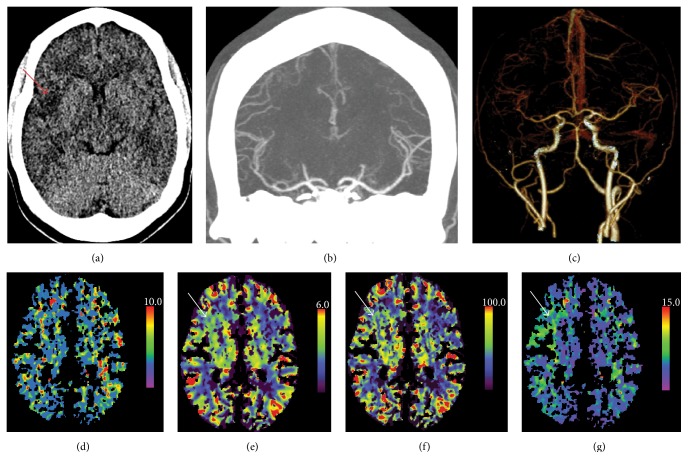
Axial unenhanced head CT scan (a) on the stroke windows (40/40 HU) demonstrated a low attenuation area in the right insular cortex (arrow). On CT angiography with coronal Minimal Intensity Projection (b) and reconstructed 3D (c) images, complete circle of Willis was identified with no gross findings of occlusion. The mean transit time (MTT) (d) showed no significant asymmetry. The cerebral blood volume (CBV) map (e) and the cerebral blood flow (CBF) map (f) demonstrated a slight asymmetry with increased CBV and CBF, respectively, in the posterior right frontal region just above the subinsular cortex extending along the anterior aspect of the subinsular cortex (arrow). The time to drain (TTD) map (g) revealed asymmetrically delayed blood drainage from the left posterior frontal region including the entire subinsular cortex (arrow). Luxury perfusion following cerebral infarction is the most likely diagnosis in the presence of changes on the unenhanced portion of the CT examination.

**Figure 2:**
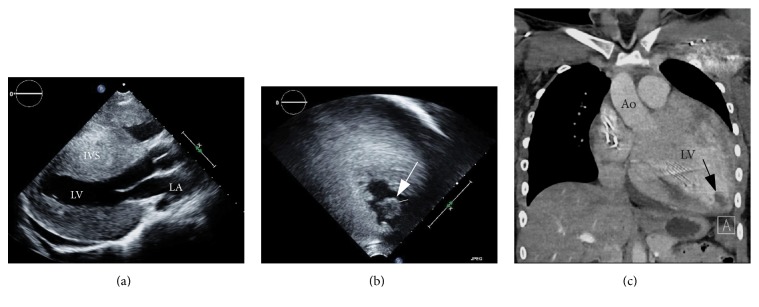
Images of hypertrophic cardiomyopathy (HCM) and a thrombus in the left ventricular (LV) apex. (a) Parasternal long axis view (a) showed severe concentric hypertrophy of interventricular septum (IVS) and LV free wall, but without an echogenic mass in the apex. (b) Off-axis apical four-chamber view identified a round echogenic mass in the LV apex measuring 1.3 × 1.4 cm (arrow). (c) A coronal reformat of postcontrast chest CT demonstrated cardiomegaly with an intracardiac thrombus at the LV apex measuring up to 1.8 cm (arrow). Ao: aorta, LA: left atrium, and LV: left ventricle.

At the most recent admission, she experienced 1-day history of reversible expressive aphasia, photophobia, and headache, followed by mild but persistent left hemiparesis. She recurrently experienced similar, but milder, episodes at home several times a week before this admission. Noncontrast head CT demonstrated an area of hypoattenuation in the right frontal lobe with hyperemia at the same site with contrast study, suggestive of enhanced reperfusion after ischemic stroke (Figure 1). CT angiography did not show any obstruction of cerebral arteries. Echocardiogram revealed unchanged severe concentric hypertrophy with normal systolic function (Table 1) but demonstrated an echogenic mass at the LV apex suggestive of intracardiac thrombus, which was also confirmed by CT scan (Figure 2). Coagulation profile was normal. She was started on continuous intravenous heparin infusion for initial anticoagulation. Her motor function recovered after intensive rehabilitation. There has been no recurrence of feeling of numbness, hemiplegia, or aphasia after she was placed on daily warfarin under strict supervision.

## 3. Discussion

Danon disease is a rare genetic muscular disorder with X-linked dominant inheritance due to mutation of lysosome-associated membrane protein 2 (LAMP2) [4, 10]. It is also commonly associated with mild skeletal myopathy and developmental delay [2, 3]. We presented a female adolescent case of Danon disease with severe concentric hypertrophy, WPW syndrome, polymorphic VT, and recurrent and progressive neurological deficit beginning with hemiplegic migraine that progressed to TIA and eventually ischemic stroke. The presence of thrombus in the LV apex was not detected until the most recent echocardiogram, which suggests its potential involvement in recurrent transient neurological deficits. Formation of intracardiac thrombus is very rare in HCM unless associated with severe LV dysfunction [11], atrial fibrillation [12], or LV apical aneurysm [13]. Izumi et al. reported a 47-year-old woman with HCM who developed thrombi in the left atrial appendage and LV apex with normal systolic function (LVEF 60.5%) and normal sinus rhythm, but she also had a history of untreated hypertension and diabetes mellitus, congestive heart failure, and recurrent stroke [14] Spinazzi et al. described 3 cases of stroke in Danon disease; all 3 had severe LV dysfunction in addition to prominent concentric hypertrophy [15]. Our patient never developed congestive heart failure, atrial fibrillation, or LV aneurysm. Marino et al. reported a 20-year-old male with neglected Danon disease who developed ischemic stroke secondary to cerebral hypoperfusion following cardiac arrest [16]. To the best of our knowledge, this is the first case of Danon disease in a young female complicated by recurrent progressive neurological deficit probably due to thromboembolic events in association with LV apical thrombus and normal LV systolic function.

This patient initially presented with infrequent episodes suggestive of hemiplegic migraine with aura but subsequently developed TIA and, finally, ischemic stroke. The relationship between initial hemiplegic migraine and ischemic stroke remains unclear [17]. Multiple head CT scans with and without contrast had been negative for stroke until the most recent event with prolonged neurological deficit when CT confirmed an ischemic stroke. Hemiplegic migraine is known to predispose to TIA and/or stroke [18]. Echocardiogram was repeatedly negative for intracardiac thrombus formation as early as 2 months prior to the incidence of ischemic stroke. It is conceivable that small microemboli might have induced transient migraine symptoms, which progressed to TIA and ischemic stroke as the size of the LV apical thrombus grew. Any new onset of neurological abnormalities in the patient with cardiomyopathy or advance heart failure warrants careful investigations for possible thromboembolism. Prompt initiation of anticoagulation is imperative in such cases. The presence of intracardiac thrombus was an unexpected finding that indicates an ominous prognosis for this patient because therapeutic options for her cardiac status were extremely limited. The presence of the LV apical thrombus was not detected by a conventional parasternal sweep or apical 4-chamber view but detected only by an off-axis view as we searched intensively due to the ischemic stroke (Figure 2). In the case of intracardiac thrombus in the left heart, aggressive anticoagulation should be initiated to prevent further thromboembolic events. It is plausible that the presence of the LV apical mass might have been missed in prior routine studies, as transthoracic echocardiogram has limited capacity in identifying intracardiac thrombus, especially that in LV apex [19, 20]. In fact, we had little suspicion of intracardiac thrombus because of her normal LV systolic function. Although heart transplant was discussed as a recommended therapeutic option [7], her history of noncompliance and lack of social support jeopardized her candidacy for this option. In children, intracardiac thrombus is a rare complication occasionally seen in patients with advanced dilated cardiomyopathy and after Fontan palliation for single ventricle and implies significant morbidity and mortality [21, 22]. The prompt diagnosis of thromboembolic events in children is challenging and requires high suspicion. Another diagnostic challenge is due to the inability to use MRI for stroke evaluation because of the implanted ICD, although this has become less important with the advent of MRI-compatible ICDs and pacemakers [23]. Limitation in the resolution of CT scan might have compromised early detection of microembolism that may be responsible for this patient's clinical presentation.

## 4. Conclusion

The presence of intracardiac thrombus indicates poor cardiovascular and cerebrovascular prognosis. We present a 17-year-old female adolescent with Danon disease who developed TIA and ischemic stroke in association with an LV apical thrombus. In this case, we encountered diagnostic challenges to recognize the presence of LV apical thrombus as a possible cause of ischemic stroke. Her neurological symptoms, including migraine headache, hemiplegia, and aphasia, resolved after initiation of aggressive anticoagulation treatment, suggesting that the presence of LV apical thrombus might have contributed to a wide spectrum of neurological deficits by distributing microemboli. Further research should be encouraged to prevent thromboembolism in cases of Danon disease and hypertrophic cardiomyopathy.

## Figures and Tables

**Table 1:** Echocardiographic assessment on the recent admission (17 years old).

	*M*-mode measurement	*Z*-score
IVSd	3.3 cm	19
LVPWd	2.2 cm	12.7
LVIDd	3.7 cm	−2.7
LVIDs	2.3 cm	−2.2
%FS	38%	0.84
%EF	76%	
LV mass	606 g	8.3

IVSd: interventricular septal thickness in diastole; LVPWd: left ventricular posterior wall thickness in diastole; LVIDd: left ventricular internal dimension in diastole; LVIDs: left ventricular internal dimension in systole; %FS: % fractional shortening; %EF: % ejection fraction.
